# Experiences with shared decision-making in psychotropic medication for people with intellectual disabilities: perspectives of experts by experience, relatives, and support professionals

**DOI:** 10.3389/fpsyt.2026.1851576

**Published:** 2026-07-03

**Authors:** Josien Jonker, Gerda de Kuijper, Evelien Ridder, Nynke Scherpbier - de Haan

**Affiliations:** 1 Department of Centre for Intellectual Disability and Psychiatry, GGZ Drenthe, Assen, Netherlands; 2 Department of Psychiatry, University Medical Centre Groningen, Groningen, Netherlands; 3 University Medical Centre Groningen, Department of Primary and Long-term Care, Research Program FOUR-C, University of Groningen, Groningen, Netherlands

**Keywords:** experts by experience, intellectual disabilities, psychotropic medication, relative and caregiver involvement, shared decision-making

## Abstract

**Introduction:**

Shared decision-making (SDM) is considered essential for person-centred care, yet individuals with intellectual disabilities (ID), their relatives, and support staff often experience barriers such as understanding complex medical information, communication challenges, and insufficient professional support. These challenges are particularly concerning in the context of psychotropic medication, which is frequently prescribed in people with ID, often without an appropriate indication, raising issues of overmedication and quality of life. Nevertheless, meaningful involvement of individuals with ID has not become routine practice. Evidence shows that both patients and carers wish to be more involved, yet little is known about how SDM is experienced in the context of psychotropic medication prescribing for people with ID. This study therefore aimed to explore how individuals with ID, their relatives, and direct support professionals perceive and experience SDM in psychotropic medication-related decisions.

**Methods:**

We conducted a qualitative interview study as part of a larger mixed-methods project on shared decision-making in medication use among people with ID. Using a phenomenological approach, we conducted semi-structured interviews with individuals with ID, their relatives, and direct support professionals to explore their experiences with SDM in the context of psychotropic prescribing. The interview guide was co-developed with experts by experience to ensure accessibility and relevance. Interviews were audio-recorded, transcribed verbatim, and analysed using thematic analysis by two researchers to enhance reflexivity and reliability.

**Results:**

The thematic analysis identified seven themes: 1) communication and relationship with clinicians, 2) understandable and tailored information provision, 3) decision-making and patient influence, 4) medication and treatment choices, 5) involvement of relatives in collaboration with professionals, 6) accessibility and continuity of care, and 7) impact on daily life and functioning.

**Discussion:**

These findings demonstrate that shared decision-making in psychotropic medication for people with ID is shaped by relational dynamics, communication quality, and organisational conditions. Meaningful involvement requires accessible and tailored information, the inclusion of relatives and direct support professionals, and care practices that support collaboration. By highlighting these needs and mechanisms, the study clarifies how SDM is experienced in this specific context and offers directions for strengthening person-centred and collaborative medication practices.

## Introduction

Shared decision-making (SDM) refers to a structured dialogue between clinicians and patients, where both parties contribute to the decision-making process by combining clinical evidence with the patient’s individual preferences, values, and life context (1). This approach has become a fundamental element of modern, person-centred healthcare, aiming to encourage mutual understanding between the treating clinician and the patient, and ensure that medical choices are tailored to what matters most to the patient (1). In recent years, SDM has progressed from a theoretical ideal to more practical models increasingly implemented across diverse healthcare settings. Research indicates that SDM not only enhances patient satisfaction and engagement but may also lead to more appropriate use of healthcare resources and improved clinical outcomes (2). Despite these benefits, the impact of SDM varies depending on the context, and its integration into routine care remains an ongoing challenge (2).

In order to understand how SDM can be effectively implemented, it is essential to consider the core components of the decision-making process. Bomhof-Roordink et al. (2019) identified 24 overarching components of SDM models across healthcare settings. Although none of the existing models are tailored specifically to individuals with intellectual disabilities (ID), insights from mental healthcare and paediatric settings provide relevant parallels. Within mental healthcare, key components include creating awareness about the existence of a choice, discussing available treatment options, deliberation, making a decision, and determining the next steps (3, 4). Paediatric SDM models similarly emphasize describing treatment options and making a decision, but also highlight the need to explore patient preferences, tailor information, determine roles within the decision-making process, and building partnership among all parties involved (3, 4). In addition, approaches in related fields have applied similar collaborative frameworks to medication decision-making in diverse populations, for example by examining mechanisms such as stakeholder involvement, treatment outcomes, and structured review processes (5, 6). Consistent with this, recent work in mental health also highlights the need for SDM models to account for relational and contextual factors as essential elements of effective SDM, such as involvement of family or social networks and discussing non-pharmacological treatment options (7).

In line with these components, the implementation of SDM in the care of individuals with ID presents specific challenges. This group often experiences barriers related to communication, cognitive functioning, and understanding complex medical information, which can limit the ability to participate meaningfully in decision-making (8). Additionally, healthcare professionals may also lack the knowledge and tools needed to facilitate an inclusive dialogue or underestimate their capacity to participate (9, 10). As a result, individuals with ID are at risk of being excluded from, or not being fully included in, decisions concerning their own health and well-being.

Although research on SDM in the context of psychotropic medication use among people with ID is still emerging, several studies highlight its critical importance. Evidence shows that psychotropic medications are frequently prescribed to this population, often off-label and in the absence of a formal psychiatric diagnosis, primarily to manage challenging behaviours (11, 12). This practice raises concerns about overmedication and reduced quality of life (13–15). Despite these concerns, meaningful involvement of individuals with ID in decisions about their medication is still not common practice. Family and paid carers, who play a key role in supporting SDM, similarly report limited involvement in medication decisions and highlight the need for approaches to strengthen SDM practices (16, 17). At the same time, studies show that both patients and carers, wish to contribute their experiences and views on treatments and be involved in decision-making (16, 18). Although SDM can improve appropriate prescribing, adherence, and greater satisfaction with care, its implementation is hindered by limited accessible information, lack of training among professionals, and assumptions about decision-making capacity (8, 10, 18–20).

To gain deeper insight into how SDM is currently experienced and perceived in the context of psychotropic medication use, we conducted a qualitative interview study involving individuals with ID, their relatives, and direct support professionals. The aim was to explore their experiences, expectations and perceived barriers and facilitators related to SDM in prescribing psychotropic medication. In addition, experiences with and evaluations of the intervention study were debated. Understanding these experiences is crucial for identifying what is needed to strengthen SDM practices and to promote more appropriate and person-centred use of psychotropic medication for people with ID.

## Materials and methods

### Ethics

The study was approved by the Medical Ethics Review Board of the University Medical Centre Groningen (2021/591 and 2022/391). All other Dutch data protection rules and research codes of conduct were adhered to as applicable. Written informed consent was obtained from all participants or their legal representatives.

### Design and participants

This interview study was conducted as a part of the mixed-methods project ‘Shared decision-making in drug treatment of mental disorders and problem behaviour in people with intellectual disabilities’. The project consisted of three components: (1) a survey investigating knowledge, expectations and current experiences regarding psychotropic medication use and SDM (18); (2) an intervention study, conducted in both a specialized mental health care setting and a primary care setting, in which satisfaction with SDM was measured before and after an intervention consisting of a structured medication review, including the provision of accessible medication leaflets and a pharmacotherapeutic treatment plan discussion with the patient and the clinician (21); and (3) this interview study.

In this qualitative interview study, we adopted a phenomenological perspective to understand how participants made sense of their experiences. This is particularly relevant when working with people with ID, as phenomenological interview studies have shown that individuals with mild to moderate ID can meaningfully describe their lived experiences, thereby offering insights into issues that matter in their daily lives (22).

Three different types of participants could be distinguished in this study, namely patients, relatives of the patient, and direct support professionals. Patients were either (1) individuals with an ID aged 16 years and older, receiving treatment at a specialized mental health care provider in the northern Netherlands and having a current prescription for a psychotropic drug, or (2) individuals with an ID, autism, and/or psychiatric and social vulnerabilities who received support from a home care organization in addition to care from their general practitioner and community pharmacist and having a current prescription for a psychotropic drug. In total, 19 participants were included: twelve patients, four relatives, and three direct support professionals. All participating individuals with ID were identified as having mild ID or similar impairments in cognitive and adaptive functioning. The mean age was 42.1 years (range 26-66). No standardised measures of intellectual or adaptive functioning were collected as part of this study. Relatives included family members who acted as informal or legal representatives. In this study, all participating relatives were mothers. Direct support professionals were healthcare professionals with experience in supporting people with ID and were affiliated with an ID care provider; all were female. All participants received a €25 gift voucher as compensation for their participation. **Table 1** provides an overview of participant characteristics in this interview study. Participant characteristics are presented in categorical form where appropriate to protect anonymity.

**Table 1:** Participant characteristics.

Participant ID	Participant type	Setting^1^	Sex
P1	Patient	MHC	Male
P2	Patient	MHC	Male
P3	Patient	MHC	Female
P4	Patient	MHC	Female
P5	Patient	MHC	Female
P6	Patient	MHC	Female
P7	Patient	MHC	Female
P8	Patient	MHC	Male
P9	Patient	PC	Female
P10	Patient	PC	Male
P11	Patient	PC	Female
P12	Patient	PC	Female
R1	Relative	MHC	Female
R2	Relative	MHC	Female
R3	Relative	MHC	Female
R4	Relative	MHC	Female
D1	Direct support professional	MHC	Female
D2	Direct support professional	MHC	Female
D3	Direct support professional	PC	Female

^1^ MHC, specialized mental health care setting; PC, primary care settin.g.

### Procedures

Participants for this interview study were recruited from all participants in the intervention study (21). Prior to the intervention study, potential participants received an information letter, an informed consent form, and an invitation for a consultation to discuss possible participation. During this consultation, both the intervention study and the interview study were explained in accessible language tailored to the participants’ level of understanding. Where applicable, legal representatives or relatives were also involved in this process. In all cases, participants were actively involved in the decision-making process, and their assent was sought and respected. At the end of the consultation, written informed consent was obtained. Participants who were legally capable provided written informed consent themselves, whereas for participants who were not legally capable, written informed consent was obtained from their legal representative. Although both studies were discussed during the same information procedure, participants could provide consent for each study independently; participation in the intervention study did not require participation in the interview study.

A semi-structured interview was developed stepwise by the research group in co-creation with experts by experience. In this study, experts by experience were defined as individuals with lived experience of having a mild intellectual disability or being closely involved as a legal representative, who are able to reflect on and contribute to improving healthcare and research based on their personal experience. This panel of experts by experience consisted of four persons with a mild ID and one legal representative. First, an initial meeting was held between the research group and the panel of experts by experience to establish a shared understanding of the purpose of the interview and to align expectations. In the second step, the research group identified relevant topics based on the current literature. These topics were then discussed with the panel of experts by experience to gather their perspectives on the listed topics and to identify any missing topics. In the third step, the research group finalized the list of topics and formulated corresponding interview questions for each topic. Next, the panel of experts by experience reviewed the questions, resulting in a revised draft of the interview. With this draft, a test interview was conducted with a volunteer with a mild intellectual disability to assess the accessibility and the understandability of the questions. The research group then made final adjustments to determine the final draft of the interview. This iterative approach contributed to the accessibility of the interview for participants with ID. The interview began with an introduction and explanation, followed by the topics participants’ contact with the clinician, changes in medication-related knowledge, and changes in the shared decision-making process, and concluded with the closing of the interview. During data collection, interviews were conducted in a flexible manner, using clear and simple language where needed. Interviewers adapted the pacing and phrasing of questions to the participants’ needs. Depending on participant preference, support persons were present during the interview.

Data were collected using audio-recorded, semi-structured interviews. Eleven of the twelve interviews were conducted face to face, and one interview was conducted via Microsoft Teams due to practical reasons. Most interviews (n=16) were conducted by the third author (ER), a psychologist and research assistant. Two interviews were conducted by an experienced ID nurse who also worked as a research assistant (n=2), and one interview by the first author (JJ), an experienced ID nurse and a PhD candidate (n=1).

### Analysis

All interviews were conducted in Dutch and were transcribed verbatim. Quotations presented in the results section were translated into English. To analyse the data, we used thematic analysis, which is compatible with descriptive phenomenology (23). We followed the six steps of thematic analysis (24): familiarisation with the data; generating initial codes; organisation of initial codes into patterns to generate themes; reviewing themes; defining/naming themes; interpretation.

During the familiarisation phase, all interviews were read multiple times by the first author (JJ). Initial codes were generated inductively, closely reflecting the participants’ experiences and perspectives. These codes were systematically organised into broader patterns and preliminary themes. Themes were reviewed and refined through an iterative process, involving comparison across transcripts, checking overlap between themes, and ensuring consistency within and between themes.

To increase the reliability of the analysis, the data were analysed independently by two researchers. First, the first author (JJ) coded all interviews, followed by coding of the second author (GdK) of a random sample of three interviews. The coding and preliminary themes were then compared and discussed. Any discrepancies or differences in interpretation were resolved through discussion until consensus was reached. To maintain reflexivity, the researchers continuously reflected on their own assumptions and interpretations, and different perspectives were openly discussed throughout the analytic process.

All conducted interviews were included in the analysis, with a focus on making full use of the dataset to capture variation and depth in participants’ perspectives. Throughout the analytic process, recurring patterns and themes were observed across interviews and stakeholder groups. Later interviews contributed to elaborate and refine existing themes. This approach enabled the development of a sufficiently rich and nuanced understanding of the phenomenon under study, providing adequate conceptual depth to address the research question, supported by the inclusion of multiple stakeholder groups and the richness of the data.

For the thematic analysis, we used ATLAS.ti (version 24).

## Results

The thematic analysis identified seven themes reflecting patients’, direct support professionals’, and relatives’ experiences, expectations and perceived barriers and facilitators related to SDM in prescribing psychotropic medication. These themes capture relational, informational, experiential, and organizational aspects, and were further elaborated in several subthemes. Participants described how communication and information provision influenced their involvement in decision-making, treatment experiences, and daily functioning. The themes are presented below and illustrated with verbatim quotations.

### Theme 1: communication and relationship with clinicians

Decision-making is grounded in the quality of the interaction between patients and clinicians. This theme reflects the conditions under which patients experience trust, a sense of safety, and can express their thoughts and concerns. Participants described how aspects of clinicians’ communication practices, attitude, and interpersonal conduct influence how comfortable and open they felt in their interaction with the clinician.

With regard to communication practices, participants mentioned the importance of asking follow-up questions, keeping agreements and reporting back.

“Because she asks further, like [name patient] tells something that she really takes time to ask a bit more in order to find out exactly what is meant.” [D3, direct support professional, PC].

“And all the things she wrote down, everything you talked about. She actually looked into all of that before the next appointment, right? And then she comes back to it.” [P10, patient, PC].

For participants, the attitude and interpersonal skills of the clinician are also important to feel comfortable in the interaction. They emphasized the importance of the clinician’s ability to listen well, take sufficient time, take the patient seriously, interact with respect, and make eye contact.

Together, the clinician’s communication practices, attitude, and interpersonal skills play a key role in helping patients feel comfortable expressing their thoughts and concerns, and in experiencing the trust needed to share their views and opinions. One of the participants described this as follows:

“Yeah, how do you say that? I don’t trust someone that quickly. And yeah, because I already know her, it’s easier to say what I have to say. If you don’t feel you can trust someone, it’s very hard to say certain things. My trust in humanity is really low.” [P5, patient, MHC].

Another participant also stressed the importance of not feeling judged:

“I think she … how do I say this? That’s a good one. Everything you experience, that you can just say it. That you don’t feel like she thinks it’s weird, for example.” [P6, patient, MHC].

### Theme 2: understandable and tailored information provision

When communication between the patient and clinician is established, it is important that the information provided is understandable and tailored to the patient. Therefore, this theme included statements in which patients describe how they experience the information they receive about their treatment and medication.

For patients, clear information is most important. For them, this means that information is as short as possible and free of difficult words and professional jargon, such as Latin terminology. As one participant mentioned:

“I do understand it, but yes, sometimes she uses words where I think, well … it doesn’t have to be that fancy.” [P4, patient, MHC].

Direct caregivers and relatives highlighted the relevance of tailored information provision. With this, they mean concrete, practical information, such as how much medication to take, how often, at what time, and what the titration or tapering process will look like. They also emphasized the need to balance the information provided, depending on what the patient can handle, and to check whether the patient has understood the information. One relative described this as follows:

“In any case, mention what you’ll be getting, how often, how much, and what it’s for. And then look at [name patient] to check if he understands it. And ask if he understands. And he always does.” [R3, relative, MHC].

### Theme 3: decision-making and patient influence

Building on patients’ experiences with communication and the clarity of information they received, this theme explores how these elements shaped the opportunities for patients to participate in decision-making. This theme covered two subthemes: (1) information as a basis for informed decision-making, and (2) providing space for personal input and preferences.

Firstly, participants described whether the information provided was sufficient, clear, and comprehensible to support well-informed treatment choices. When participants felt they had sufficient information to make a decision, they particularly emphasized the importance of clarity. This referred to the structured way in which appointments were organized within the intervention, the supportive information provided through the easy-to-read leaflets, and the use of clear explanations in accessible language. When discussing the structure and course of the intervention with the interviewer, one participant mentioned:

“Yes, I think so. Everything is clearer, everything is nicer, everything is more organized.” [P1, patient, MHC].

Another participant similarly underlined the need for clarity, explaining:

“I knew enough about it, but some things could have been clearer. It could have been clearer in the sense that it could have been shorter instead of long-winded. I mean, the explanation of the plan, because it was a lot of information. It works better when it is much clearer and when you explain it calmly, without very complicated Dutch. Just not that highly formal Dutch.” [P4, patient, MHC].

Relatives echoed this need for clarity. They found it helpful to review information together once more. Together with the involvement of multiple disciplines, this provided a sense of reassurance and contributed to participants feeling better informed. One relative described this as follows:

“Well, you know, you go in with an open mind. And you know which medication she uses, and by now you’ve learned about the side effects and so on. But having everything explained together at that moment is really clarifying. One person explains it one way, the pharmacist explains it differently from the nurse, and very differently from the doctor. And that complements each other so nicely. I actually like involving others in shared decision-making. It’s a kind of reassurance. You hear the pros and cons from three different sides, and what is best for her. And that combination feels very reassuring.” [R4, relative, MHC].

Moreover, participants reported the extent to which they felt able to express their own views and preferences. Most participants reported that they were able to express their views or had learned to do so over the past years. They also described that discussions about their medication more and more felt like a joint process. Others still found it challenging to express their opinion, yet they tried to do so or sought support from someone they trusted. Having real influence and making truly shared decisions was a new experience for several participants. As one participant explained:

“Just how she thinks about it herself (the prescribing physician) and how I think about it too. We realized together that it’s helpful medication for me, and that she made the right decision in giving it to me.” [P2, patient, MHC].

Another participant emphasized the importance of having a say and being able to explore concerns openly with the clinician:

“That I also have a say in whether I want the medication or not. She thinks along with me and asks why I don’t want it. Then it turns out there’s fear, or that a similar medication made me trip before. But then we look together and see it’s different. In the end, we decide together whether we do it or not. I feel free to give my opinion. Nothing is pushed on me.” [P7, patient, MHC].

For some participants, this was the first time they had experienced such a collaborative decision-making process:

“It felt special. At that moment you’re not a number. You’re really a patient who gets to help decide about medication, and I found that very pleasant.” [P2, patient, PC].

This view was echoed by a relative:

“Normally the doctor decides about medication. Never the pharmacist, never just the nurse. But now it was all together. You can discuss things carefully, ask questions, and get a conclusion from all three perspectives. That was very nice.” [R1, relative, MHC].

### Theme 4: medication and treatment choices

In this theme, the focus lies on patients’ experiences with the practical aspects of their medication use and treatment options. Participants reflected on how they understood the purpose and effects of their medication, whether changes or adjustments to their treatment were made, and whether alternative treatment options were discussed with them or not.

Participants described the impact of medication on their behaviour, mood, and emotional stability. Several participants emphasized the importance of staying stable or expressed fears of experiencing a relapse if their medication were to change.

“No, not really. What I do notice is that I stay stable now, with this stuff. Yes, I still call it ‘stuff’, but well, it is what it is.

Interviewer: It sounds like it’s very different from how it used to be, that you weren’t that stable back then?

No, messing around for almost four years with medication, you know. And also, those courses of medication, for four years continuously, one after the other. And those courses make you very unstable too, not to forget. And when you then have medication for the psychological or mental things, and you also have a course on top of it, that works both ways in a negative way. Because the one … uh … suppresses the effect of the other, for example. And that is what I kept running into.” [P4, patient, MHC].

Having few or no side effects was experienced as highly positive, whereas others reported concerns about existing side effects or anxiety about the possibility of developing side effects. Participants also noted that interactions with other substances, such as alcohol or combinations of medications, could influence how they felt or functioned. Most participants also indicated that alternative treatment options were not discussed, or that such discussions were limited to alternative pharmacological treatments.

Also, one relative highlighted that there is little insight into the long-term consequences of ongoing medication use, raising concerns about how this may affect the patients’ life.

“If it’s used for too long, what are the consequences? I don’t know. For example, after five years or so, you know, what does it do to the body? How will the side effects then influence her life? After five years, or would there maybe be a new agent by then that might be better, with fewer side effects? We don’t know that, of course.” [R1, relative, MHC].

Finally, one participant also emphasized the importance of reviewing treatment on time.

“And it also needs to be evaluated in a timely way, because that was my own point…. I thought, well, that actually happens very little. But I also became aware of that again just by being here. It just keeps standing there for years, right? When someone is stable.” [D1, direct support professional, MHC].

### Theme 5: involvement of relatives in collaboration with professionals

This theme explores how relatives and professional caregivers were involved in patients’ medication-related treatment appointments and decision-making processes. Participants described several ways in which relatives supported patients during appointments, ranging from providing practical or emotional support to speaking on behalf of the patient or helping them make sense of treatment information. One participant explained how the presence of a relative helped him to process information:

“Sometimes when I hear things, I don’t understand them, or my head gets too full, and then I don’t remember it anymore. So it’s really helpful to have an extra pair of ears.” [P6, patient, MHC].

A direct support professional described how they actively helped clarify treatment information:

“When I noticed that for [name patient] the information was a bit complicated for her or that she became a bit nervous, then I asked her, ‘Is it clear?’. And if she said it wasn’t clear, I tried to explain what was meant and then checked with the clinician if that was correct.” [D3, direct support professional, PC].

Beyond these forms of support, participants also reflected on the position and influence of relatives, and whether their views were invited and acknowledged. One of the relatives illustrated this as follows:

“When [name clinician] doesn’t have everything clear, he checks back what he thinks was said and then looks at me like ‘Is that correct?’. So I can add to it, and he is happy with that.” [R3, relative, MHC].

However, confidentiality sometimes limited the involvement of relatives, as one of the participants explained:

“There are certain questions that … that people around me are not allowed to know. Things that are a bit sensitive, you know. I don’t dare to tell [name clinician] that. Not because of [name clinician] as a person, but because she also has contact with my husband, and he must not know.” [P1, patient, MHC].

In addition, participants also reflected on how collaboration between professionals and those surrounding the patient took shape. They described experiences of professional alignment and interprofessional collaboration, sometimes across different services or sectors.

“We had contacts with several services and complex-case meetings, because we couldn’t solve it without their help. And what really makes a difference is that active collaboration between clinicians, because sharing knowledge is exactly what you need to get out of such complex situations.” [R1, relative, MHC].

### Theme 6: accessibility and continuity of care

This theme captures how patients experienced the accessibility of their healthcare providers and the continuity of their treatment. Participants described how easy or difficult it was to reach clinicians, and whether they felt supported when questions or problems arose. Participants reported on different aspects, such as the ability to contact them by phone or to attend timely appointments, as well as the regularity, availability, and predictability of follow-up appointments.

Most participants reported being satisfied with the frequency of their appointments, with only a small number expressing a desire for more frequent contact. Participants also highlighted several aspects that were important to them regarding the accessibility and continuity of care. In terms of accessibility, they appreciated being able to contact their clinician by email, valued receiving a prompt return call after leaving a message, and noted that scheduling treatment sessions could sometimes be challenging due to the clinician’s full agenda. With respect to continuity of care, participants emphasized the importance of having a substitute clinician available in cases of illness and suggested that scheduling treatment appointments in advance may help prevent crisis situations.

“Well, that’s kind of the thing, it’s often in a crisis situation. So maybe, if [name patient] had more balanced, regular appointments, then you wouldn’t only call when there’s a crisis.” [D1, direct support professional, MHC].

### Theme 7: impact on daily life and functioning

The final theme explores how participants experienced the broader impact of patients’ symptoms, treatment, and treatment-related decisions on daily life and overall functioning. Participants described how changes in behaviour, mood, or energy shaped their ability to manage daily living, and how treatment outcomes influenced their sense of well-being.

In addition, participants shared concerns, hopes, and expectations about their future, illustrating how experiences across the earlier themes ultimately translated into emotional, social, and practical consequences.

Both patients and relatives emphasized that appropriate medication plays a key role in enabling a life worth living, in which individuals can engage in the everyday activities that matter to them with a sense of enjoyment.

“I want my life to feel more ‘liveable’, to have more interest in things. I am a bit more ‘liveable’ now, but still, I don’t feel interested in anything. I don’t feel like doing anything. I don’t even feel like going to the store; I just don’t feel like anything.” [P3, patient, MHC].

Relatives echoed this view, expressing the wish for their child to feel well and to be able to do their usual activities. They described a threshold of functioning that needs to be met for life to feel acceptable.

“We are happy with where we are now. I wish him much more, because he is awake for about ten hours a day, and spends seven or eight of those on the couch. So his life is very limited. In hindsight, he went through multiple severe depressions during the time he was doing so badly………. His quality of life was so bad for a period that I thought this can’t go on much longer…………. And I say, I still find it very limited, but I do have peace with this, with how his life looks now.” [R1, relative, MHC].

Future perspectives were linked to the limitations patients experience due to their mental health problems. Participants expressed a need to discuss their questions, wishes, and expectations regarding the future, such as their desire to have children and their future support needs.

## Discussion

In this qualitative interview study, we examined how individuals with ID, their relatives, and direct support professionals experience and perceive SDM in the context of psychotropic medication prescribing. The findings highlight a broad set of relational, informational and organisational factors that affect SDM. These insights show that communication processes, tailored information provision, the collaborative involvement of relatives and direct support professionals, and the organisation of care shape participants’ sense of meaningful involvement in medication decisions, their everyday experiences with medication treatment, and the perceived impact on quality of life and future implications.

The seven themes identified in this study can be understood within the building components of established SDM models. In line with the systematic review of Bomhof-Roordink et al. (2019), our themes and subthemes reflect several components of generic SDM models as identified in their analysis. These include, for example, building a trusting relationship, tailoring and providing information, exploring preferences, and deliberation leading to joint decision-making. Such components closely align with our themes concerning communication and relationship with clinicians, understandable and tailored information provision, decision-making and patient influence, and medication and treatment choices. Consistent with paediatric and mental health SDM models, participants also highlighted elements that enable genuine partnership and what is needed for such partnership, as well as the importance of involving relatives and direct support professionals. Thereby, the reported impact on daily life and functioning stresses the need to embed relational and contextual determinants, including family and professional carer involvement, into SDM practice (16–18, 25, 26). Together, these insights offer conceptual refinement to existing SDM frameworks by showing how multiple determinants converge in the specific context of ID and mental health care.

Focusing on SDM in psychotropic medication treatment in people with ID, our findings also resonate with the three overarching themes identified by Sheehan et al. (2019), respectively medication beliefs and experiences, carer role, and decisional processes. In our study, participants likewise emphasised how lived experiences with psychotropic medication shaped their expectations and preferences. Moreover, our themes concerning the involvement of relatives and direct support professionals, as well as communication and relationships with clinicians, echo Sheehan et al.’s characterisation of the carer role as both interpretative and mediating with treatment discussions. Finally, the decisional processes described by Sheehan et al. (2019), including struggles, negotiations, and relational tensions in appointments with clinicians, show parallels with our findings on communication, the extent to which participants felt heard, and the organisational conditions that enable or constrain meaningful SDM.

Taken together and building on the points above, our findings further demonstrate that SDM in psychotropic medication treatment for people with ID is not solely a procedural or informational task, but is deeply embedded in relationships, everyday caregiving practices, and organisational aspects. These experiences emphasize not only the significance of relatives and direct support professionals in the care of people with intellectual disabilities, but also the complex interplay between informal support networks and the broader network of professionals involved in treatment and treatment decisions. Part of this complexity lies in the need for interdisciplinary collaboration, where divergent professional perspectives must be aligned, and information must be shared across organisational boundaries to support decision-making. In practice, however, this may be difficult due to potential reluctance of physicians to collaborate on psychotropic medication changes and due to the involvement of multiple specialties in prescribing, which can fragment responsibility (27). Another issue concerns confidentiality practices, which, while essential for safeguarding privacy, can hinder the flow of relevant information exchange and complicate the involvement of relatives or direct support professionals in discussion about SDM, as illustrated by a participant who felt unable to share sensitive information because the clinician was also in contact with a family member (P1, patient). Previous research similarly shows that people with ID and their carers do not receive the essential information they need, which limits their opportunity to participate meaningfully in medication decisions (16, 18).

### Strengths and limitations

The strength of this study lies in the diversity of participants and the tailored interview design. We interviewed 19 participants across two different care settings, which allowed us to capture a broad range of experiences. By including people with ID, their relatives, and direct support professionals, we were able to collect perspectives from diverse stakeholders in different settings and gain a more complete picture of SDM. A second strength was that experts by experience contributed to the development of the semi-structured interview guide. Their input helped to ensure that the questions were accessible and relevant for people with ID. Finally, the study adds valuable knowledge to an area where research is still very limited, especially regarding SDM in psychotropic medication treatment for people with ID.

This study has several limitations. First, selection bias is likely, as participants were recruited from those who had already taken part in the intervention study, which may overrepresent individuals who are more engaged with the topic. Therefore, the generalizability of the findings is limited due to the specific sample and context. Additionally, this study was conducted within the Dutch healthcare context, and differences in healthcare systems across countries, such as the roles of prescribers, access to specialised mental healthcare services, and funding structures, may influence how SDM is implemented in other settings. These contextual factors should be considered when interpreting the findings. Furthermore, the findings regarding the SDM process in medication use are limited to just psychotropic medication use. Fourth, because multiple interviewers conducted interviews, subtle differences in style and probing may have influenced the data. However, they worked closely as part of the same project team and followed a fixed interview format. A further consideration is that the influence of behavioural presentation during appointments was not explicitly explored. While aspects related to communication, trust, and feeling heard and understood were reflected in the findings, more immediate behaviour during consultations such as agitation or distress were not specifically examined. These factors may influence clinician decisions and the process of shared decision-making and could be explored further in future research. Finally, given the target group of people with ID, socially desirable responding and restricted depth on some topics cannot be ruled out.

### Impact

The findings of this study have several important implications for practice. By showing how people with ID, their relatives, and direct support professionals experience SDM around psychotropic medication, the results of the study highlight concrete areas where communication, collaboration, and care organisation can be strengthened. Especially the need for more accessible information, clearer coordination among professionals, and systematic involvement of relatives and support staff in medication decisions are important issues. The results also underline the significance of creating space for the expression of lived experiences of people with ID, including their views on medication effects and everyday functioning. Therefore, the results of this study can support services in shaping more person-centred and collaborative medication practices, and provide direction for developing training, interdisciplinary routines, and organisational policies that better facilitate SDM for people with ID. Summarizing, this study clearly suggests that implementing SDM for people with ID requires not only procedural clarity around options and decisions, but also sustained attention to relational dynamics, contextual factors, and the practical organisation of care.

## Conclusion

This study shows that SDM in psychotropic medication for people with ID is shaped by relationships, everyday caregiving practices, and organisational conditions. Our themes align with established SDM models and echo findings from earlier qualitative research in the UK examining lived experiences with psychotropic medication, carer roles, and decision-making processes. Together, these insights underline that SDM extends far beyond providing information or presenting options. Meaningful patient involvement requires accessible communication and tailored information, the inclusion of their relatives and direct support professionals, and organisational routines that facilitate collaboration. By connecting these elements to the lived experiences of people with ID, this study extends existing SDM frameworks by showing how they manifest in this specific care context, and by deepening understanding of needs and mechanisms relevant to this context. In this way, this study adds valuable insight to a still underexplored area of healthcare, clarifying what SDM means for people with ID in the context of psychotropic medication.

## Data Availability

The raw data supporting the conclusions of this article will be made available by the authors, without undue reservation.
